# TECHNOLOGY-SPECIFIC AGEISM AS A PREDICTOR OF NOT OFFERING TECHNOLOGY-BASED PHYSIOTHERAPY FOR OLDER ADULTS

**DOI:** 10.1093/geroni/igad104.3720

**Published:** 2023-12-21

**Authors:** Ittay Mannheim, Cynthia Neiertz, Eveline Wouters

**Affiliations:** Ben Gurion University of the Negev, Beer-Sheva, HaDarom, Israel; Fontys University of Applied Science, Eindhoven, Noord-Brabant, Netherlands; Fontys University of Applied Science, Eindhoven, Noord-Brabant, Netherlands

## Abstract

Incorporating digital technologies in healthcare can potentially improve the quality and accessibility of older persons’ care. However, ageist attitudes among healthcare professionals can lead to discrimination in treatment decisions and deprive older patients of technology-based treatment. This study explores whether technology-specific ageism influenced physiotherapists’ decisions to use technology-based healthcare with older patients. Seventy-eight physiotherapists in Luxembourg filled out an online survey. Technology-specific ageism was measured by the Attitudes Towards Older Adults Using Technology (ATOAUT-11) scale. Additional measures included the Expectations Regarding Aging scale, attitudes towards technology use in the work environment, and whether physiotherapists had not offered technology-based treatment in the past because of a patient’s age. Thirty-one percent of the participants reported that they had not offered technology-based treatment to a patient in the past because of the patient’s older age. Importantly, using logistic regression, it was found that higher levels of technology-specific ageism predicted not offering technology-based treatment, such that participants with more negative attitudes (1 standard deviation) were two times more likely not to offer treatment. Positive attitudes toward using technology in the work environment were also found to be a significant predictor. All other characteristics (gender, age, work experience, and percentage of patients over 50) did not predict not offering treatment. This study provides initial evidence that technology-specific ageism may lead to discrimination and deprive older persons of receiving optimal treatment. More research is needed to identify the magnitude of ageism in using technology-based treatment in healthcare and develop interventions to mitigate it.

